# Supplementary material for: Mutations in pregnancy‐associated plasma protein A2 cause short stature due to low IGF‐I availability
Source: EMBO Mol Med. 2016 Feb 22;8(4):363–74. doi: 10.15252/emmm.201506106 (PMC4818753; doi:10.15252/emmm.201506106)
Supplement: Supplementary file 1 — Appendix [file EMMM-8-363-s001.pdf]

APPENDIX

Mutations in pregnancy-associated plasma protein A2 cause short stature due to low IGF-I availability

[1]Andrew Dauber, M.D., MMSc, [2]María T. Muñoz-Calvo, M.D., Ph.D., [2]Vicente Barrios, Ph.D., [3]Horacio M. Domené, Ph.D., [4]Soren Kloverpris, Ph.D., [5]Clara Serra-Juhé, Ph.D., [6]Vardhini Desikan, M.D., [2]Jesús Pozo, M.D., Ph.D., [7]Radhika Muzumdar, M.D., [2]Gabriel Á. Martos-Moreno, M.D., Ph.D., [8]Federico Hawkins, M.D., Ph.D., [3]Héctor G. Jasper, M.D., [9]Cheryl A. Conover, Ph.D, [10]Jan Frystyk, M.D., Ph.D., DMSc [11]Shoshana Yakar, Ph.D., [1]Vivian Hwa, Ph.D., [2]Julie A. Chowen, Ph.D., [4]Claus Oxvig, Ph.D., [12]Ron G. Rosenfeld, M.D. [5]Luis A. Pérez-Jurado, M.D., Ph.D., [2,*]Jesús Argente, M.D., Ph.D.

Institutions: [1]Cincinnati Center for Growth Disorders, Division of Endocrinology, Cincinnati Children's Hospital Medical Center. Cincinnati, OH, 45229 USA; [2]Department of Pediatrics & Pediatric Endocrinology. Hospital Infantil Universitario Niño Jesús. Instituto de Investigación La Princesa. Universidad Autónoma de Madrid. Department of Pediatrics. CIBEROBN. Instituto de Salud Carlos III. Madrid, 28009 Spain; [3]Centro de Investigaciones Endocrinológicas "Dr. César Bergadá" (CEDIE), CONICET, FEI, División de Endocrinología, Hospital de Niños Ricardo Gutiérrez, C1425EFD Buenos Aires, Argentina; [4]Department of Molecular Biology and Genetics, Aarhus University. Aarhus, 8000 Denmark; [5]Genetics Unit, Universitat Pompeu Fabra, Hospital del Mar Research Institute (IMIM) & CIBERER. Instituto de Salud Carlos III. Barcelona, 08003 Spain. [6]Department of Pediatrics, Division of Pediatric Endocrinology, New York Medical College, Valhalla, NY, 10595 USA; [7]Division of Endocrinology, Children's Hospital of Pittsburgh. Pittsburgh, PA, 15224 USA; [8]Department of Endocrinology. Hospital Universitario 12 de Octubre. Universidad Complutense de Madrid. Madrid, 28041 Spain; [9]Division of Endocrinology, Mayo Clinic, Rochester, MN, 55905 USA; [10]Medical Research Laboratory, Department of Clinical Medicine, Faculty of Health, Aarhus University and Department of Endocrinology and Internal Medicine, Aarhus University Hospital. Aarhus, 8000 Denmark. [11]Department of Basic Science and Craniofacial Biology, New York University College of Dentistry. New York, NY, 10010 USA; [12]Oregon Health and Science University. Portland, OR, 97239-3098 and STAT5 LLC, Los Altos, CA, 94022 USA.

Content:

Appendix Figures S1-S2………………………………………………………………………………….. **2-3**

Appendix Figure legends…………………………………………………………………………………. **4**

Appendix Tables S1-S4……………………………………………………………………………………………… **5-8**

Family 1

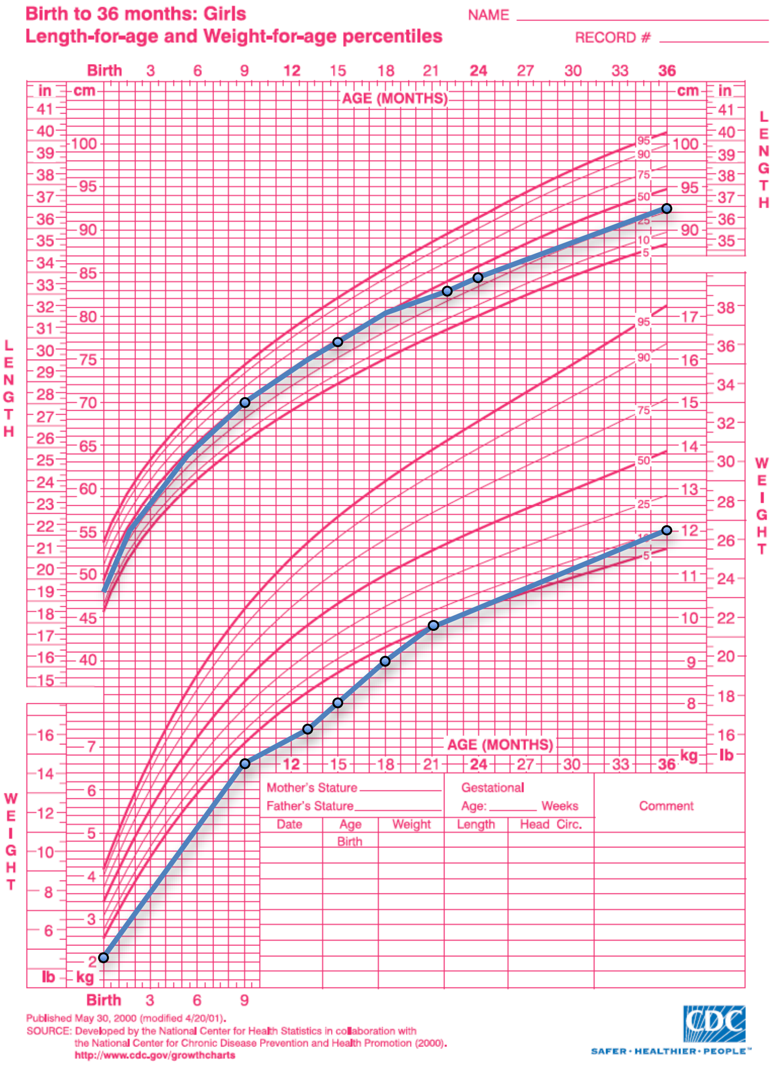

Subject II.1

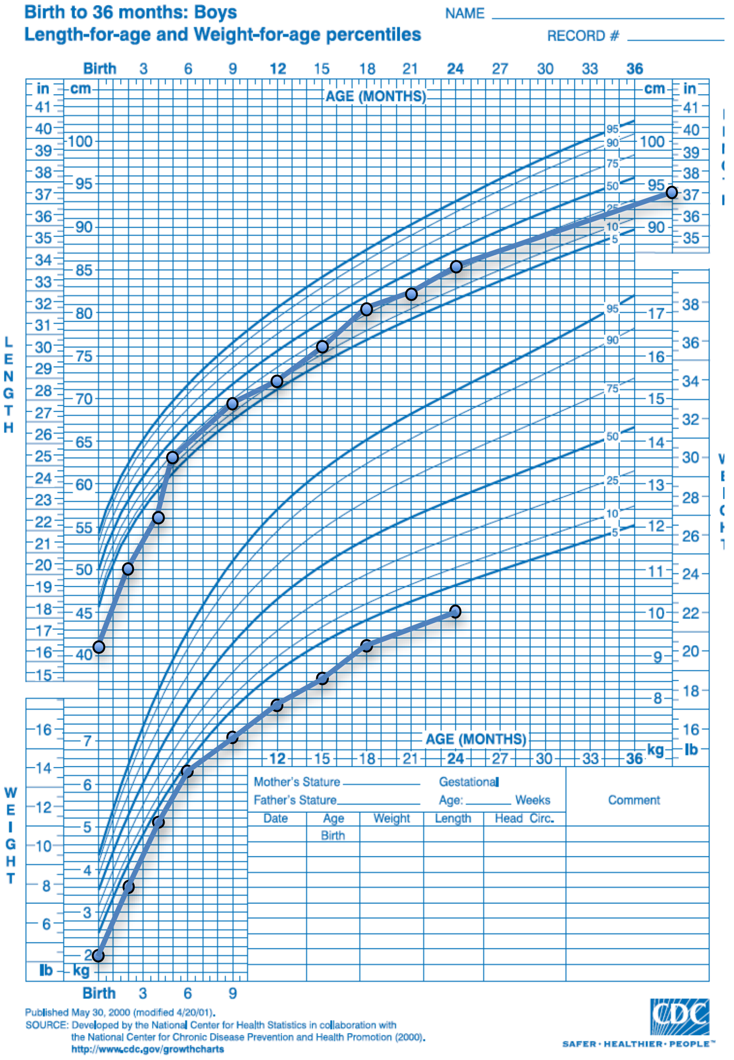

Subject II.3

Appendix Figure S1

A

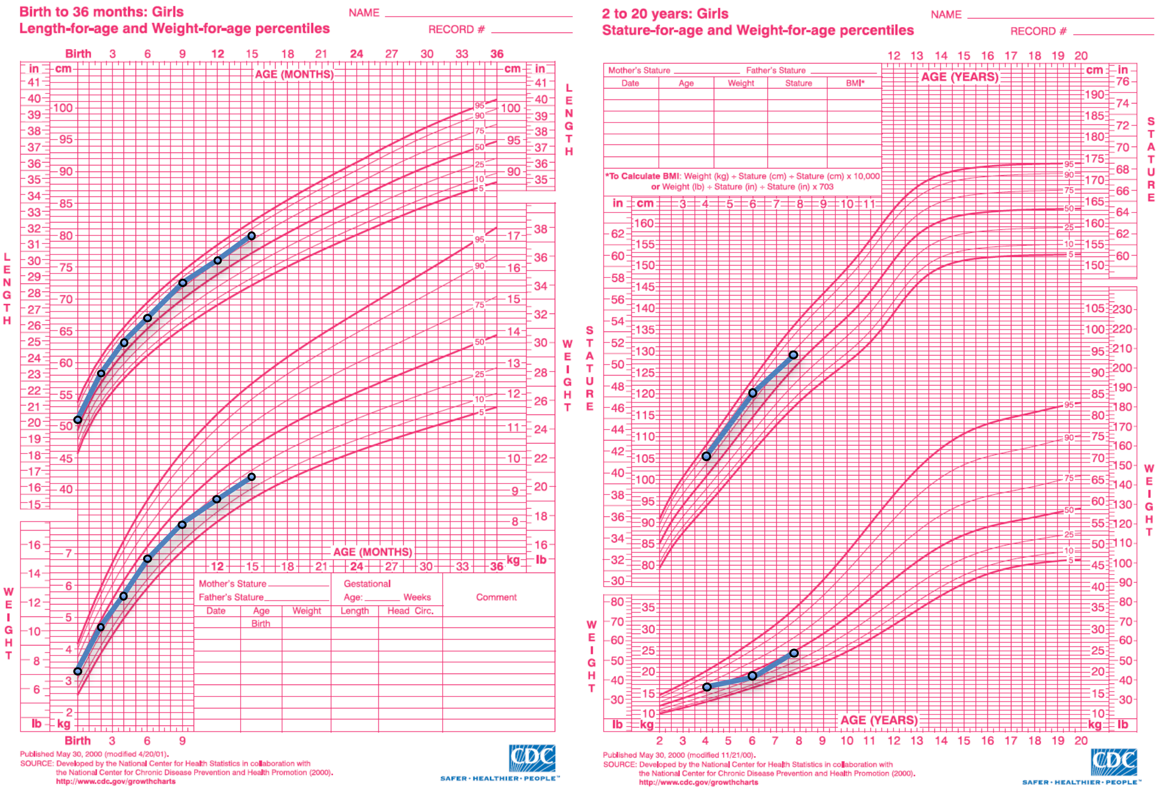

Subject II.2

B

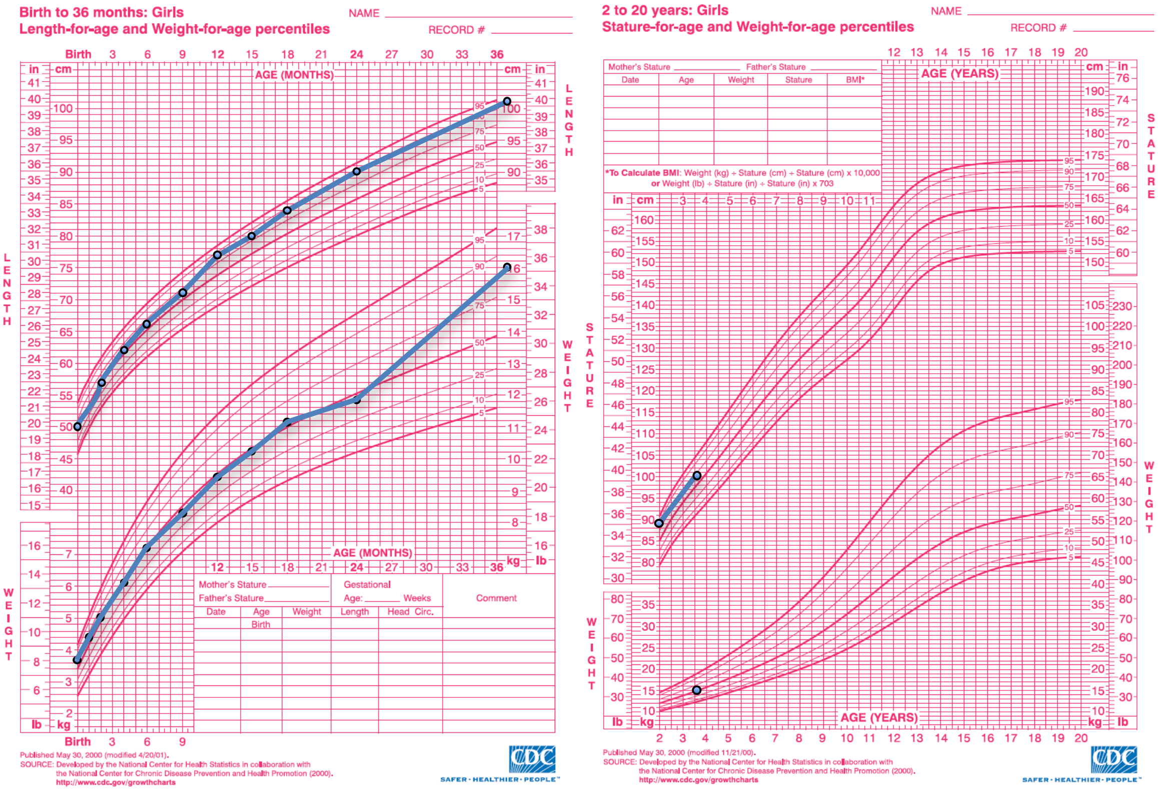

Subject II.4

Appendix Figure S2

Figure Legends

<u>Appendix Figure S1</u>: Growth Charts from Birth to 36 Months of Subjects II.1 and II.3 from Family 1.

<u>Appendix Figure S2</u>: Growth Charts from Unaffected Siblings of Family 1. A. Subject II.2. B. Subject II.4.

<u>Appendix Table S1</u>: Longitudinal IGF-I and IGFBP-3 Levels in Subject II.3 from Family 2

Age (years, months)	IGF-I (ng/ml)	IGFBP-3 (ng/mL)
6y10m	517.3	
7y5m	534.3	
7y7m	545.6	6400
8y6m	726.6	
9y0m	698.3	
10y10m	519	7300
11y5m	564	
11y10m	786	
12y11m	702	6400
13y2m	825	3700
14y0m	895	8700
15y8m	1031	8100
18y	1060	

<u>Appendix Table S2</u>: Candidate Genes Based on Exome Sequencing Analysis in Family 1

Chr.	Position	Reference allele	Variant allele	Genotype	Gene	cDNA change	Protein change	Minor allele frequency
1	158152680	C	T	Homozygous	CD1D	NM_001766 c.C620T	A207V	Novel
1	158651382	G	A	Homozygous	SPTA1	NM_003126 c.C466T	R156W	0.0001243
1	161021465	G	C	Homozygous	ARHGAP30	NM_001025598 c.C1059G	S353R	0.0000182
1	176564667	_	insAT	Homozygous	PAPPA2	NM_020318 c.1927_1928insAT	D643fs25*	Novel
10	134699337	G	A	Heterozygous	TTC40	NM_001200049 c.C3431T	T1144M	0.0002229
10	134738299	G	A	Heterozygous	TTC40	NM_001200049 c.C1157T	T386M	0.0001469

Chr. – Chromosome.
Minor allele frequency was taken from the ExAC Browser http://exac.broadinstitute.org

Appendix Table S3: Regions of homozygosity in Family 2

Chromosome	Start Position	End Position	Length (MB)
1	171755170	178019772	6.3
3	186205930	194753890	8.5
8	54308719	61809918	7.5
13	74096953	84845106	10.7

Appendix Table S4: Total and Free IGF-I Levels in *Pappa2* Knock Out Mice (16 weeks of age)

	**Total IGF-I (ng/ml)**	**Free IGF-I (ng/ml)**
Male Wild Type (N=9)	28 ± 2.6	5.7 ± 0.98
Male Knock Out (N=8)	44 ± 1.4	0.2 ± 0.08
P-value for comparison between groups	0.0001	0.0012
Female Wild Type (N=12)	27 ± 2.3	6.6 ± 1.40
Female Knock Out (N=7)	43 ± 3.7	0.3 ± 0.17
P-value for comparison between groups	<0.0001	<0.0001
