# Supplementary material for: Mutations in pregnancy‐associated plasma protein A2 cause short stature due to low IGF‐I availability
Source: EMBO Mol Med. 2016 Feb 22;8(4):363–74. doi: 10.15252/emmm.201506106 (PMC4818753; doi:10.15252/emmm.201506106)
Supplement: Supplementary file 2 — Expanded View Figures PDF [file EMMM-8-363-s002.pdf]

Expanded View Figures

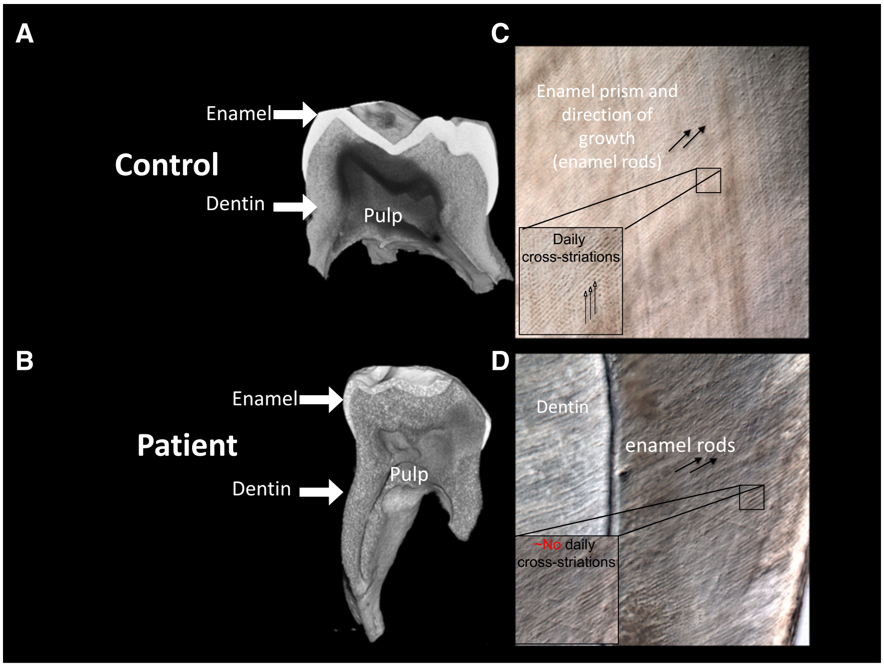

Figure EV1. Enamel and dentin layers in the patient have low mineral density.

Three-dimensional micro-CT analysis of extracted tooth from Subject II.1 in Family 1.

A, B Micro-CT analyses of the control (A) and the patient (B) teeth show reduced enamel and dentin mineral density, suggesting that odontoblasts, which lay dentin, and amyloblast, which lay enamel, exhibit impaired mineralization.

C Polarized light imaging of the enamel layer in control subject exhibit a prism-like structure, composed of enamel rods showing the direction of enamel growth and the daily cross striations, reflecting the daily activity of amyloblasts, which lay the mineral.

D Polarized light imaging of the enamel layer in the patient tooth, no distinct daily cross striations lines were found, suggesting that amyloblast activity is impaired and that mineral is not laid appropriately.

© 2016 The Authors

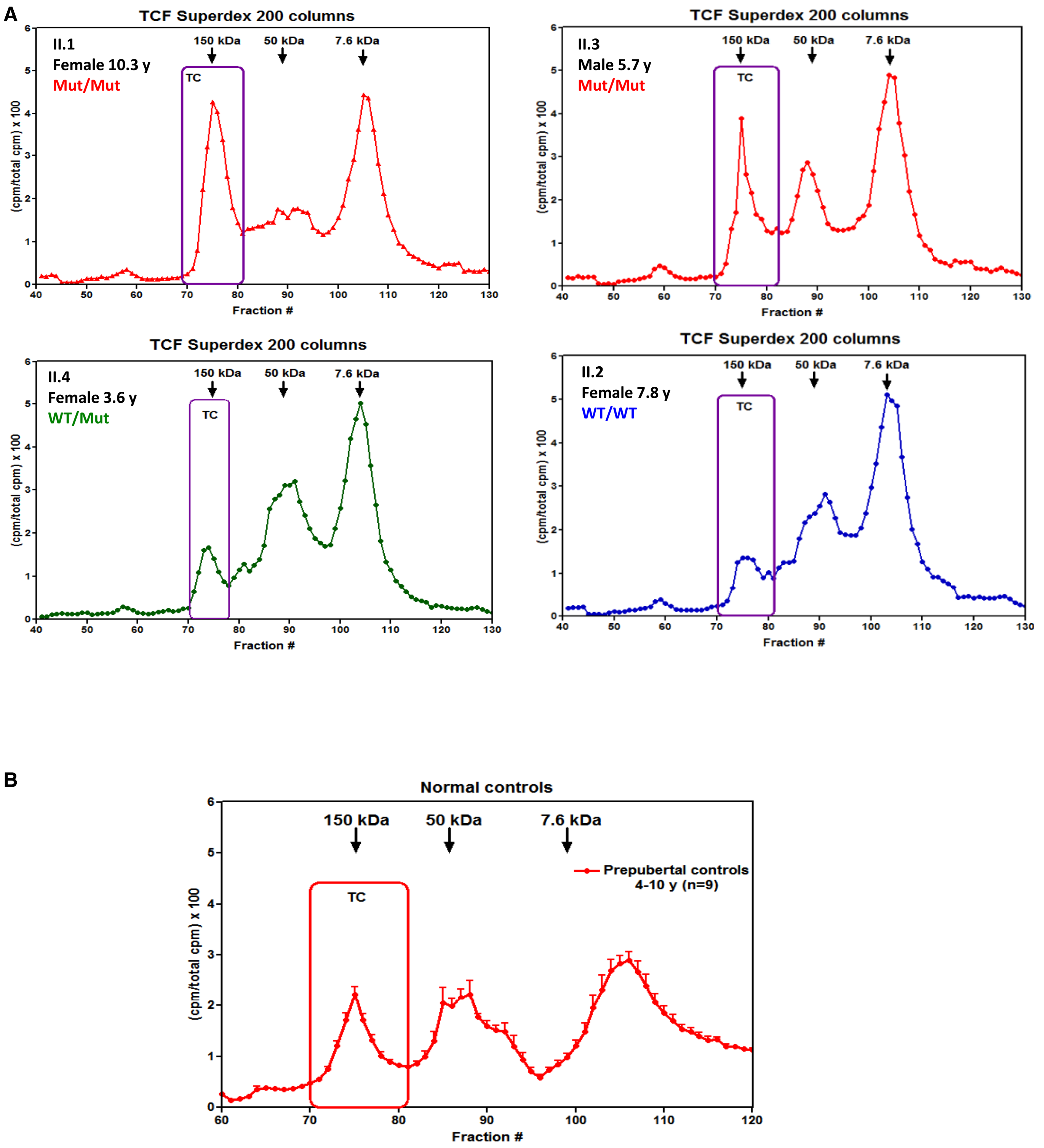

Figure EV2. Analysis of capacity to form IGF-I complexes.

A, B (A) Ternary complex (TC) formation followed by size-exclusion column chromatography. The purple box corresponds to the TC peak formed by exogenous IGF-I and endogenous acid labile subunit (ALS) and IGF-binding protein (IGFBP) -3 or -5. The homozygous affected subjects (II.1 and II.3) had an elevated capacity to form TC compared to their heterozygous (II.4) and homozygous wild-type (II.2) siblings. This suggests that the ability to form ternary complexes is significantly elevated in the patients homozygous for the *PAPPA2* mutation and this is most likely due to their increased concentrations of circulating ALS, IGFBP-3, and IGFBP-5. The ability to bind IGF-I in the ternary complex was also higher in the homozygous patients compared to nonrelated prepubertal controls shown in (B). Mean ± SEM. $n = 9$.

 © 2016 The Authors
